# Mitral isthmus ablation using a circular mapping catheter positioned in the left atrial appendage as a reference for conduction block

**DOI:** 10.18632/oncotarget.17092

**Published:** 2017-04-13

**Authors:** Takahiko Nishiyama, Takehiro Kimura, Taishi Fujisawa, Kazuaki Nakajima, Akira Kunitomi, Shin Kashimura, Yoshinori Katsumata, Nobuhiro Nishiyama, Yoshiyasu Aizawa, Keiichi Fukuda, Seiji Takatsuki

**Affiliations:** ^1^ Department of Cardiology, Keio University School of Medicine, Shinjuku-ku, Tokyo, 160-8582, Japan

**Keywords:** catheter ablation, perimitral atrial flutter, mitral isthmus, artial fibrillation, steerable sheath

## Abstract

**Background:**

For perimitral atrial flutter (PMFL) developing after catheter ablation of atrial fibrillation (AF), to create a complete conduction block at the mitral isthmus (MI) is mandatory to terminate it, however, it is still challenging.

**Methods:**

This study consisted of 80 patients (74 male, 61 ± 8.1 years) undergoing MI ablation. After a circular mapping catheter was positioned at the neck of the left atrial appendage (LAA), the MI ablation was performed on the MI line just below the LAA neck targeting the earliest activation recording site of the LAA catheter during pacing from the coronary sinus (CS). When ablation during CS pacing was not successful, an RF delivery during LAA pacing was applied targeting the earliest activation site just below the MI line. If the endocardial approach failed, an RF application inside the CS was attempted.

**Results:**

With the endocardial approach, acute success was achieved in 51/80 patients (64%). Additional epicardial ablation from the CS was performed in 26/29 (90%) endocardially unsuccessful patients and conduction block at the MI was achieved in 21/26 (81%). Overall, complete conduction block at the MI was achieved in 72/80 patients (90%). At a mean follow-up of 16 ± 6 months, 20 patients (25%) had recurrence of atrial arrhythmias (AT: 12, AF: 8), and 10 (AT: 7, AF : 3) underwent a second procedure in which an LMI block line was completed in 3 (33%). PMFL was diagnosed in 6 out of 7 AT patients. No complications were observed.

**Conclusions:**

Creating linear lesions just beneath the neck of the LAA was highly successful under the guidance of a circular mapping catheter in the LAA using a steerable sheath. An RF application from the CS was needed in less than half of the cases.

## INTRODUCTION

Atrial fibrillation (AF) is associated with an increased risk of a stroke, heart failure, and all-cause mortality [1–3]. Catheter ablation is a standard non-pharmacological management for symptomatic AF resistant to pharmacological therapy [4–6]. To encircle the pulmonary veins (PVs) is a highly effective therapy for patients with AF [7, 8]. A possible problem after catheter ablation of AF is the development of a left atrial tachycardia (AT), mostly related to reentry including the mitral isthmus (MI) [9, 10], that is, perimitral atrial flutter (PMFL). PMFL does not respond well to antiarrhythmic drug treatment and frequently requires repeat procedures [11, 12]. However, the completion of the ablation line at the MI may be difficult to achieve and may often require epicardial ablation from the coronary sinus (CS) [13–15]. Catheter ablation of the MI is one of the most challenging procedures after PV isolation and an incomplete block line may lead to the recurrence of PMFL [16].

Recent studies have reported that steerable sheaths have significantly improved the success rate of MI ablation [17, 18]. In these studies, MI ablation procedures were performed with a maximum power of 40W to 50W. Higher the radiofrequency (RF) power increased the risk of complications such as cardiac tamponade and an occlusion of the CS [19]. Further, the procedures were performed using a linear ablation technique under anatomic and electrogram guidance [20]. The catheter was positioned at the ventricular edge of the lateral mitral annulus, and then the sheath and catheter were rotated clockwise to the left inferior pulmonary vein (LIPV). If the initial attempt failed to create complete bidirectional block, mapping was performed along the line to detect any gaps or a different line was created in a more lateral position. In that conventional technique, to map the gap along the line might often be difficult because the local electrocardiograms can be obscured due to tissue edema.

When the circular mapping catheter is positioned at the neck of the left atrial appendage (LAA), the lateral side of the mapping catheter will be positioned parallel to the MI line if it is positioned just beneath the LAA. Further, constant pacing from the CS could tell us the position of the conduction gap along the MI line. Hence, we investigated the feasibility of making linear lesions along the MI just beneath the neck of the LAA under guidance using the activation sequence of the LAA ostium.

## RESULTS

### Patient characteristics

The baseline clinical characteristics of the 80 included patients are summarized in Table 1. The patients consisted of 20 with paroxysmal AF (25%), 41 with persistent AF (51%) and 19 with longstanding persistent AF (24%). In the persistent AF and longstanding persistent AF patients, 59/60 (98%) underwent a box isolation. All patients with paroxysmal AF and one with persistent AF underwent a circumferential PV isolation, and 7/21 (33%) patients underwent an additional roof line ablation. All PVs were successfully isolated, but dormant PV conduction was induced in 13/80 (16%) patients and treated by an additional ablation. Bidirectional conduction block of the CTI was achieved in all patients.

**Table 1:** Baseline characteristics

Total N = 80			
Age	61 ± 8.1	E (cm/s)	77 ± 16
Male (n)	76	A (cm/s)	57 ± 29
Height (cm)	170 ± 7.0	Dct (ms)	179 ± 49
Body Weight (kg)	73 ± 10	E’ IVS	8.7 ± 2.5
Paroxysmal AF	20	E’ LW	12.3 ± 3.5
Persistent AF	41	TEE LAA-E (cm/s)	39 ± 17
Longstanding persistent AF	19	TEE LAA-F (cm/s)	44 ± 19
Duration of AF (M)	46 ± 41	Cr (mg/dl)	1.0 ± 0.2
CHADAS_2_ score	0.8 ± 1.0	BNP (pg/ml)	147 ± 149
LVEDD (cm)	4.8 ± 0.5	Number of AAD	0.3 ± 0.6
LVESD (cm)	3.1 ± 0.6	Number of β-blocker	0.5 ± 0.5
Left atrial diameter (cm)	4.3 ± 0.6		

Data are given as mean ± SD. LVEDD = Left ventricular end-diastolic diameter, LVESD = Left ventricular end-systolic diameter, E = Mitral flow E-wave, A = Mitral flow A-wave, Dct = E-wave deceleration time, E’ IVS= Tissue Doppler of the septal mitral anular velocity, E’ LW = Tissue Doppler of the lateral mitral annular velocity, TEE = transesophageal echocardiogram, LAA-E = left atrial appendage emptying flow velocity; LAA-F = left atrial appendage filling flow velocity

### Procedural results

**Figure 1:**
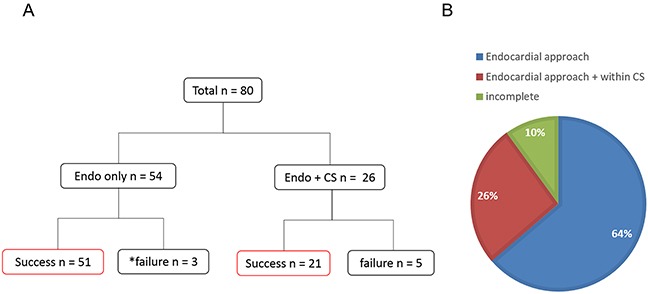
The acute procedural outcome is shown In **(A)** the MI block-line was achieved in 51/80 patients with only endocardial ablation. An epicardial ablation from the CS was performed in 26/29 unsuccessful patients. Complete conduction block along the MI was achieved in 21/26. * No epicardial ablation was performed because of a high impedance or inappropriate potentials. In **(B)** the percentage of a complete and incomplete line of block of the MI is shown.

The procedures consist of circumferential and box PV isolation were achieved in all patients. Acute success was achieved in 51/80 patients (64%) with an exclusive endocardial approach. Additional epicardial ablation from the CS was performed in 26 out of 29 endocardially unsuccessful patients (90%) and conduction block at the MI was achieved in 21 patients (81%) (Figure 1). The reasons for avoiding an epicardial ablation from the CS in the other three patients were no earliest activation recorded in the CS in two cases and a high impedance recorded in the CS in one. Overall, complete conduction block at the MI was achieved in 72/80 patients (90%). No complications were observed in the included patients.

### Anatomical characteristics and ablation energy

The results of the anatomical characteristics are shown in Table 2. The length and maximum myocardial thickness of the MI were similar between the 3 groups. The prevalence of a pouch at the MI was similar. An interposition of the branch of the left circumflex artery (LCX) was significantly more common in Group 3 (Group 1 vs. 2 vs. 3 was 31% vs. 48% vs. 75%, respectively, P < 0.05). The distance to the CS or LCX from the LA did not differ. Table 3 shows the comparison of the ablation data in each group. The total RF duration and amount of energy deliveries significantly differed among the three groups. A block line of the MI was completed with the shortest RF time and fewest RF energy deliveries in Group 1. The RF applications inside the CS did not differ between Group 2 and Group 3.

**Table 2:** Charactersitics of the mitral isthmus by computed tomography

	Group 1 (N = 51)	Group 2 (N = 21)	Group 3 (N = 8)	p
Length of MI (mm)	36 [31–39]	34 [32–37]	36 [34–37]	n.s
Maximum thickness (mm)	3.3 [2.9-3.7]	3.2 [2.5-3.5]	3.0 [2.6-3.2]	n.s
Pouch morphology	6%	14%	25%	n.s
Interposed cicumflex	31%	48%	75%	<0.05
Distance to CS (mm)	3.1 [2.8-4.2]	3.2 [2.4-4.1]	4.2 [3.4-4.6]	n.s
Distance to LCX (mm)	3.2 [2.6-4.6]	2.6 [2.3-4.7]	2.5 [2.3-3.7]	n.s

Data are shown as the median and interquartile range and relative frequencies.

MI = mitral ishmus, CS = coronary sinus, LCX = left coronary circumflex

**Table 3:** Comparison of the ablation data of the mitral isthmus

	Group 1 (N = 51)	Group 2 (N = 21)	Group 3 (N = 8)	p
Endocardial ablation time (min)	6.8 [5.1-10.4]	16.7 [12.4–22.3]	31.0 [27.2-36.8]	<0.05
Endocardial ablation energy (J)	13461 [9879–18660]	31171 [23083–43263]	62456 [51930–74423]	<0.05
CS ablation time (min)	-	3.0 [1.6-3.9]	2.6 [1.2-3.5]	n.s
CS ablation energy (J)	-	3898 [2078–5277]	3350 [1640–4751]	n.s
Total ablation time (min)	6.8 [5.1-10.4]	20.9 [16.6-24.9]	34.5 [28.0-40.2]	<0.05
Total ablation energy (J)	13461 [9879–18660]	35580 [29303–45563]	67091 [52738–79009]	<0.05

Data are shown as the median and interquartile range.

CS = coronary sinus

### Long term follow up

At a mean follow-up period of 16 ± 6 months, a total of 20 out of 80 patients (25%) had recurrence of atrial arrhythmias which consisted of 12 ATs (Group 1 vs. 2 vs. 3 was 7 (14%) vs. 4(19%) vs. 1 (13%), p=NS), and 8 (Group 1 vs. 2 vs. 3 was 3 (6%) vs. 4 (19%) vs. 1 (13%), p=NS) AF episodes.

**Figure 2:**
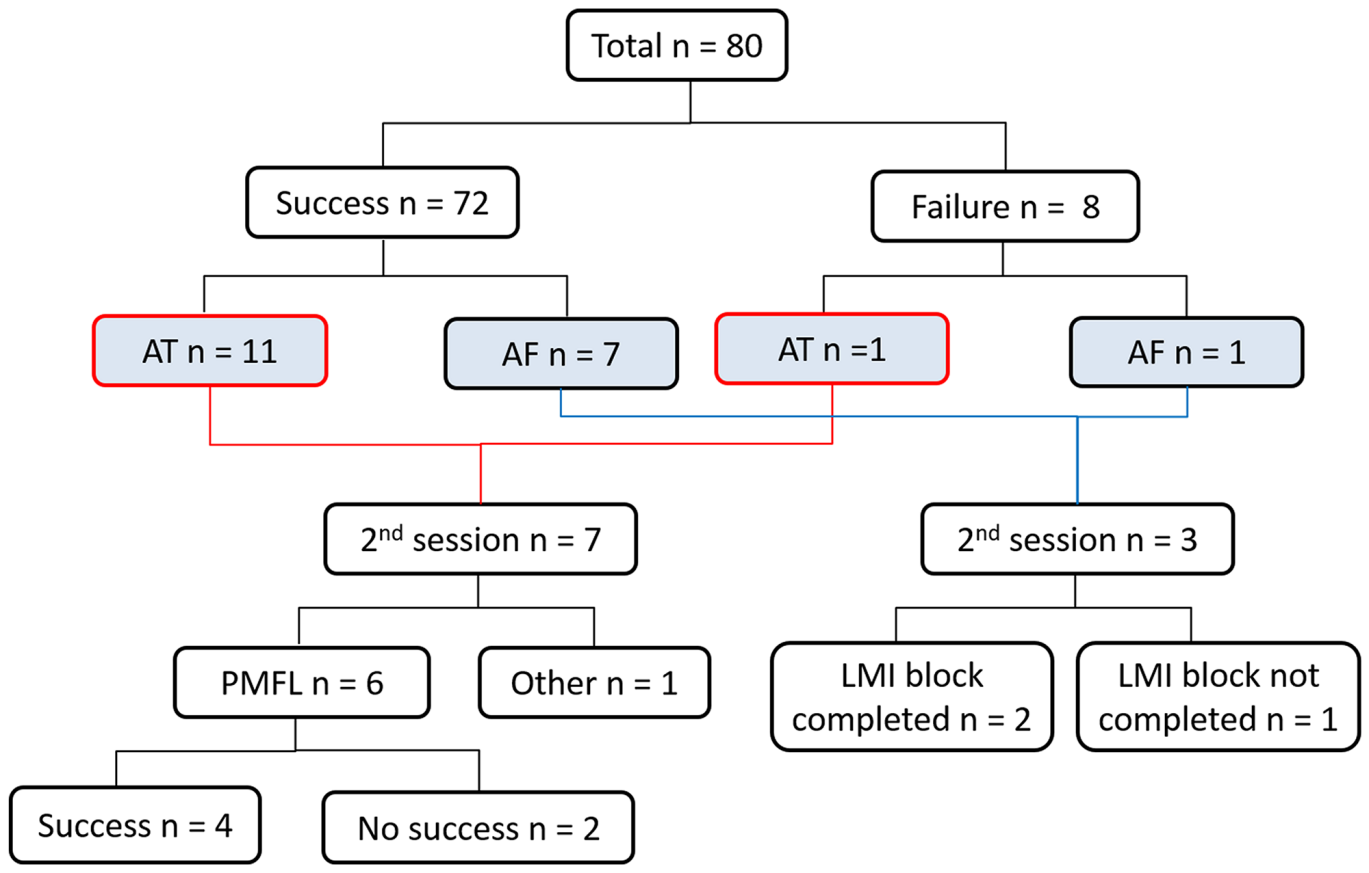
The clinical outcome is shown Nineteen patients had recurrence of atrial arrhythmias (AT: N = 11, AF: N = 8). Among them, ten (AT: N = 7, AF : N = 3) underwent a second procedure. PMFL was observed in 6 out of 7 AT patients. In two cases, a VOM ethanol infusion was performed. Follow up period: 16 ± 6 months. VOM: vein of Marshall.

Ten patients including two patients with incomplete block of the MI during the 1^st^ session (ATs :7, AFs: 3) underwent a second procedure. All ATs were diagnosed by an activation map constructed with the CARTO system and by an entrainment mapping technique, which revealed a PMFL in 6 out of 7 patients. Those were treated by an RF application at the gaps along the MI line. In 2 patients, however, complete conduction block at the MI was not accomplished by the RF applications (Figure 2). In the patients who underwent a 2nd procedure, a complete MI block line was observed only in 3 out of 10 patients (33%).

**Figure 3:**
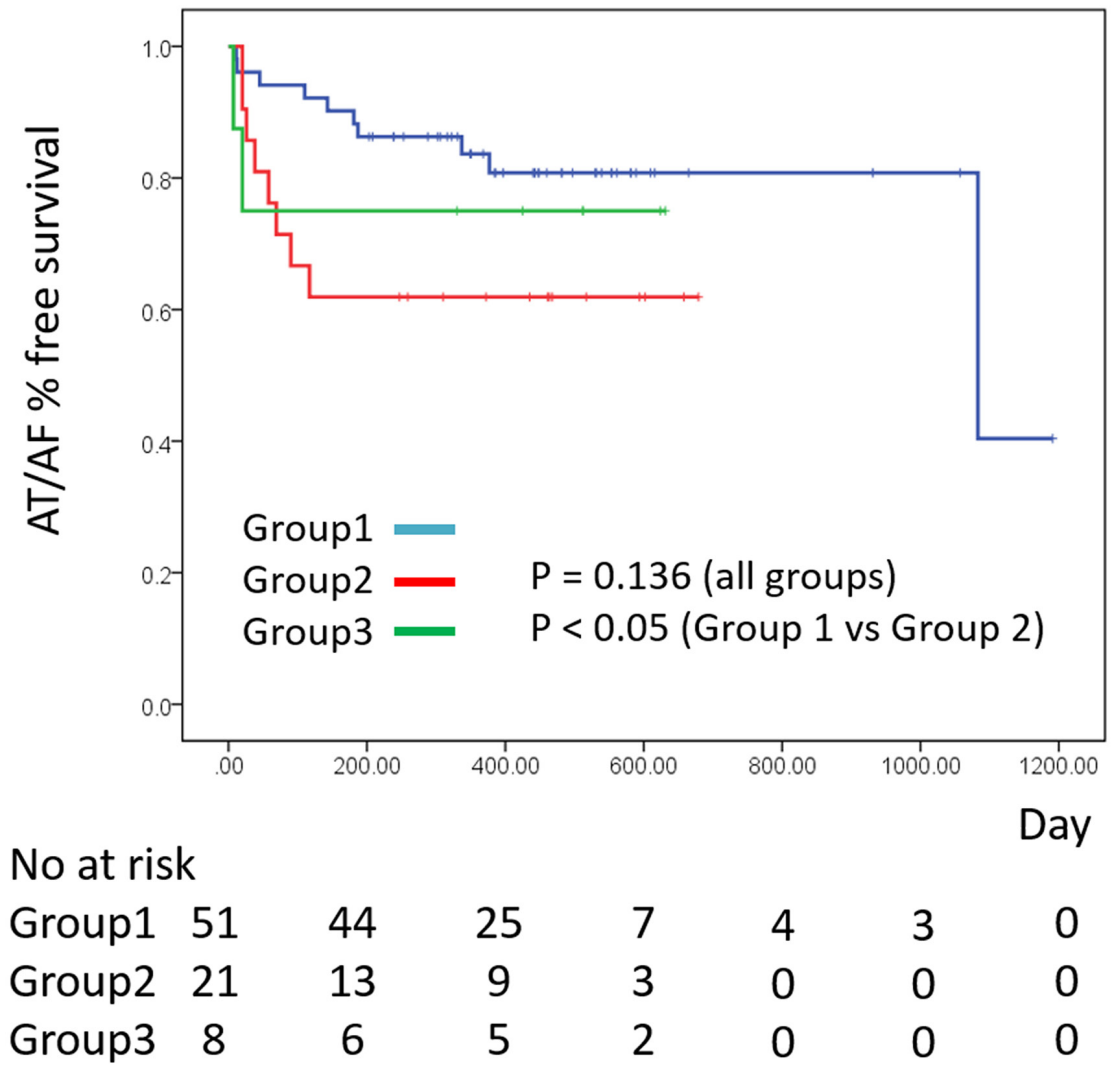
Kaplan-Meier curves of the AT/AF free survival rate for each group

The Kaplan-Meier curves of the AT/AF free survival rate for each group are shown in Figure 3. The rate of recurrence among all groups was not statistically significant. However, the rate of recurrence in Group 2 was significantly higher than that in Group 1 (P < 0.05).

## DISCUSSION

Linear ablation is an important strategy for avoiding macro-reentrant AT after catheter ablation of AF [13, 21]. Creation of a complete block of the MI remains challenging despite using irrigated-tip catheters and 3D electroanatomical mapping systems [22]. In recent studies, success rates of an MI block line range from 65% to 92% [13, 17, 23, 24]. Because an incomplete block was reported to increase the risk of PMFL, it is important that complete electrical block of the MI is achieved [16].

In this study, creating linear lesions at the MI under guidance using the activation sequence in the LAA during pacing from the CS safely demonstrated a high success rate of bidirectional conduction block. The ablation power was restricted up to 35 W to avoid any serious complications such as steam pops and cardiac tamponade. Notably, our approach could reduce the need for epicardial RF applications from within the CS. We successfully completed the MI line block in 64% with only an endocardial approach, whereas more than two-thirds of the patients required RF ablation within the CS to create complete conduction block of the MI in a previous study [15].

As factors associated with a successful MI ablation, previous studies suggested the importance of the steerable sheath and contact force [17]. Anatomical factors such as the myocardial thickness and presence of pouches, length of the MI and distance to the LCX, high take off of the LIPV, and connection of the VOM have also been reported [25–28]. In this study, an interposition of the LCX was observed significantly more often in the group with an incomplete line of block.

In our approach, the block line of the MI was positioned at a more superior level than that of the conventional approach. The myocardium at the superior level of the left lateral ridge is thicker than that at the inferior level, so the creation of transmural lesions might be difficult [29]. Especially, the ridge in the LMI area is thicker than the other areas in the LA [30]. In addition, the VOM was traced on the epicardial aspect of the ridge, because the VOM might disturb the creation of the transmural lesions.

Regarding the CS, the line of conduction block at the superior level, ie. at the distal CS, can be easier to create than that at the proximal CS, since the distal CS can be narrower and has less blood flow than the proximal CS. In other words, a transmural lesion might be completed easily in the distal CS, and our approach was valid for reducing the number of RF applications inside the CS [31].

The other alternative approach which was not affected by the ridge and cooling effect of the CS was an anterior block line. However, this approach would provoke a significant delay in the LAA activation. The systolic timing of the LAA could coincide with that of the ventricle because of delayed conduction of the lateral LA. As a result, the function of the LAA decreases and the incidence of a cerebral infarction increases due to thrombosis [32]. In our approach, the conduction time to the LAA was not delayed because the conduction through Bachmann's bundle was preserved, and the LAA function might not have been influenced.

In addition, we would like to place emphasis on the guidance using the activation sequence of the circular catheter. The potentials in the LAA effectively support the ablation of the MI because the circular mapping catheter was positioned relatively parallel to the MI line. In contrast, because the electrode catheter in the CS during pacing from the LAA was positioned vertically on the MI line, the sequence could not precisely show the earliest site.

In ten patients who underwent repeat sessions, recovery of the conduction of the LMI was observed in 67% in our study. The recurrence during the follow up unfortunately was relatively high. The risk of PMFL was high if a complete block line was not achieved during the first procedure. Recovery of the bidirectional block was present in 61-73% of the patients during the repeat ablation procedures in the previous reports [16, 33]. The patients without any recurrence of AF or AT were not restudied. Therefore, the true incidence of recovered conduction of the LMI may not have been estimated accurately in all patients.

Although the reason for the higher incidence for recovery of conduction at the MI as compared with the cavotricuspid isthmus is not clear, the several reasons are considered for the reconnection of the posterior line. Anatomical factors such as the myocardial thickness and the presence of pouches, length of the MI and distance to the LCX, and connection of the VOM have also been reported. Previous studies demonstrated that atrial myocardium thickness around the MI, and that the depth of the pouch may be the important factors in achieving and maintaining bidirectional block [34]. In addition, it also recently has been shown that when the LCX artery courses between the MI and the CS. The LCX artery may act as a heat sink preventing the development of a transmural lesion [26].

The results of our study suggest an interposed LCX is predictive of unsuccessful ablation at the MI. This finding suggests that the blood flow prevents a transmural lesion. An additional ablation into the CS may increase the risk of arterial injury. The CT imaging may be helpful to reduce the risk of complications. If an interposed LCX is detected on CT imaging, the ablation at the MI should be avoided. Because it may increase the risk of AT related to incomplete lesions. An anterior line may be an alternation approach, but the risk of LAA isolation is considered as mentioned.

### Study limitations

The limitation of this study was the retrospective nature and relatively small group of patients. The success was influenced by the operator's experience. During the follow up, this study showed the maintenance of the MI block line in patients who underwent repeat ablation procedures.

## MATERIALS AND METHODS

### Study population

This study was approved by our Institutional Review Board based on the ethical guidelines of the Declaration of Helsinki. All patients provided their written informed consent before the procedure. Eighty patients (74 males, 61 ± 8.1 years, persistent AF: 41, longstanding persistent AF: 19) with drug-refractory AF undergoing catheter ablation for a circumferential or box PV isolation followed by an MI ablation at a single center were included in this study. Persistent AF was defined as AF sustaining beyond 7 days, or lasting <7 days but necessitating pharmacological or electrical cardioversion. Longstanding persistent AF was defined as continuous AF of a >1 year duration. All patients underwent electrocardiography (ECG) gated cardiac multidetecter computed tomography (MDCT) (GE Healthcare, Amersham Place, England) before the ablation therapy. Oral anticoagulation with warfarin or non-Vitamin K dependent oral-anticoagulants was performed for at least 1 month before the procedure. Warfarin was adjusted for a target prothrombin time international normalized ratio (PT-INR) of 2.0-3.0, and intravenous heparin was used in the perioperative period if the PT-INR was less than 2.0 as necessary. Transesophageal echocardiography was performed within 48 hours before the procedure to exclude any left atrial (LA) thrombi. All antiarrhythmic medications were discontinued for at least 5 half-lives before the ablation.

### Analysis of the anatomical mitral isthmus

MDCT imaging performed prior to the ablation was analyzed in the late systolic phase images using a workstation (AW Server 2.0, GE Healthcare, Amersham Place, England). The mitral isthmus was defined as the region between the ostium of the LIPV and lateral mitral annulus. The diameters of the anatomical structures and distance to the adjacent blood vessels were measured referring to a previous study [26].

### Catheter ablation for the PV isolation

An electrophysiologic study was performed with the patients under deep sedation and monitored by a Bispectral Index monitor (Aspect Medical Systems, Newton, MA, USA). An oral airway and facial mask for the auto servo ventilation (ASV) (ResMed, Teijin, Tokyo, Japan) to stabilize the respirations was provided in all patients. The surface ECG and intracardiac electrograms (filtered from 50 – 300 Hz) were stored on a computer-based labsystem (Labsystem Pro, Bard Ep, Lowell, MA, USA). From the right jugular vein, a 14 polar electrode catheter was introduced into the CS, of which the proximal electrodes covered the high right atrium and the superior vena cava. The distal electrode of the catheter placed in the CS was inserted to the 2 - 3 o’clock position along the mitral annulus in the 55° left anterior oblique radiographic projection. From the right femoral vein, two or three long sheaths were introduced into the LA. A 3.5 mm saline-irrigated mapping ablation catheter (Navi-Star Thermocool SF, Biosense Webster, Diamond Bar, CA, USA) was inserted into the LA and utilized through a steerable sheath (Agilis sheath, St. Jude Medical, Minneapolis, MN, USA). The PV isolation procedure was performed under the guidance of the CARTO system (Biosense Webster, Diamond Bar, CA, USA) using the image integration of the LA from the MDCT. A single circular catheter for an ipsilateral PV isolation and double circular catheters (LASSO 2515 NAVeco variable catheter, Biosense Webster, Diamond Bar, CA, USA and Inquiry AFocus, St. Jude Medical, Minneapolis, MN, USA) for a box isolation were employed. RF energy was delivered with 30-35W with 8 or 15 ml/min, and 20-25 W with 8 ml/min at the posterior wall near the esophagus. The complete lesion of PV isolation was confirmed that there was no dormant conduction by the injection of an adenosine triphosphate. Further, we inserted an esophageal thermosensor (Sensi Therm, St. Jude Medical, Minneapolis, MN, USA) to avoid any esophageal injury and cut the RF delivery when the esophageal temperature reached 38°C.

### Mitral isthmus ablation

**Figure 4:**
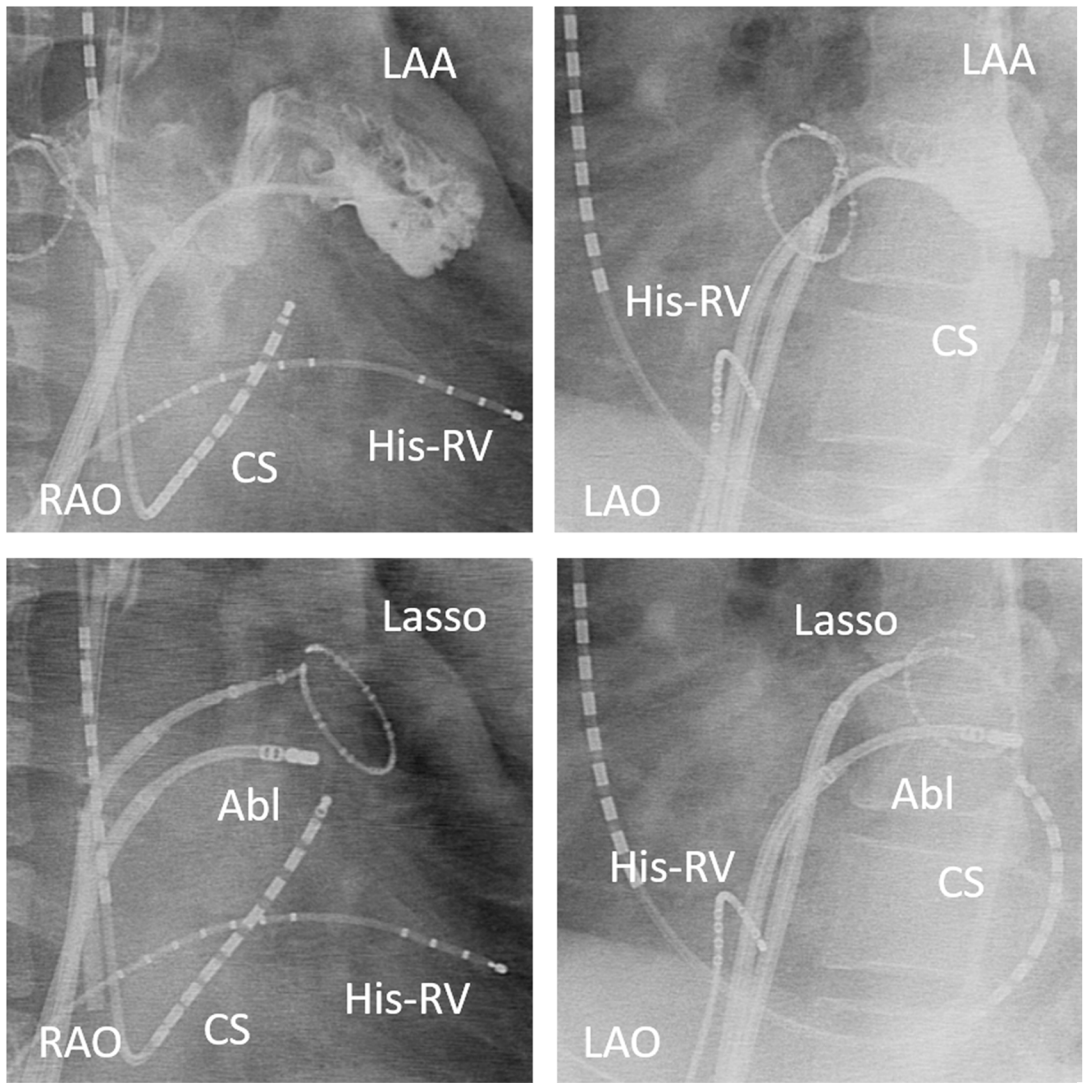
Angiography of the left atrial appendage (LAA) showing the boundary between the LAA and lateral free wall (upper panel) Radiography showing the circular mapping catheter in the left atrial appendage, and right anterior oblique (RAO) and left anterior oblique (LAO) views of the catheter position during the mitral isthmus ablation (lower panel). The ablation was started with the ablation catheter located at the earliest activation site during CS pacing. A steerable sheath was used to facilitate the catheter manipulation.

**Figure 5:**
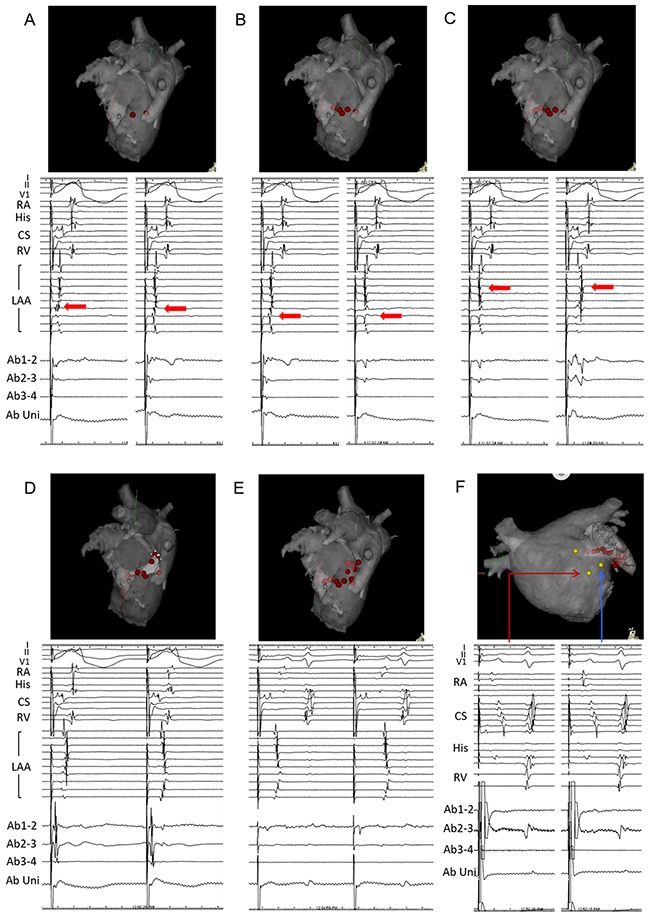
Progressive changes in the LAA sequence were observed along the mitral isthmus ablation line during CS pacing In (A)-(D) we mapped the earliest activation site, which preceded the sequence of the LAA. Intracardiac recordings during pacing from the distal pole of the CS catheter are shown in the lower panel (left panel: pre ablation, right panel: post change in the sequence). In E, the MI block-line was completed. In F, conduction block on the opposite side was demonstrated by differential pacing from the upper side of the ablation line. An anatomical description of the 3D mapping system is shown in the upper panel.

Patients with an induced PMFL after the PV isolation or longstanding persistent AF, regardless of any PMFL induction, underwent an MI ablation in this study. After confirmation of the boundary between the LAA and lateral free wall by angiography of the LAA, we positioned a circular mapping catheter at the neck of the LAA during pacing from the CS and mapped the earliest activation site preceding the sequence of the LAA (Figure 4). An RF application was targeted at just below the earliest activation site of the LAA circular catheter electrodes. The ablation applications was delivered one after another targeting the earliest activation along the line (Figure 5). When complete conduction block during CS pacing could not be achieved, an RF delivery during LAA pacing was applied targeting the earliest atrial activation site below the MI line. After the initial line failed to achieve the conduction block, ablation was performed slightly anterior and posterior positions. If the endocardial approach failed to create conduction block at the MI, an epicardial ablation inside the CS was attempted (25W, 8 ml/min irrigation rate). The ablation catheter was advanced into the distal CS to the level of the endocardial lesion. The catheter was gradually withdrawn to the mid level of CS while early potential was being estimated. If conduction block was not completed, the procedure was repeated for a maximum of five attempts. The RF energy delivery was cut-off if the temperature reached 43°C. Complete bidirectional conduction block of the MI line was confirmed by the differential pacing technique reported previously [13].

### Comparison between the patients with and without an epicardial ablation from the CS and the patients with an incomplete block line

We classified the patients with complete block of the MI into 2 groups as follows according to the approach: only enodocardial (Group 1) and endocardial plus epicardial (Group 2). The patients with an incomplete block line of the MI were defined as the unsuccessful group (Group 3). We compared the anatomical characteristics of the MI, ablation energy, and clinical outcome among these three groups.

### Follow-up

The patients were hospitalized for 2–4 days for an optimal anticoagulation after the procedure. After discharge, the patients were followed-up at 1 month from the procedure and every 3 months by a cardiologist as a routine follow-up. A portable tele-ECG (Cardiophone, Nihon Kohden, Inc., Tokyo, Japan) was used to detect any recurrence in all patients and 24-hour Holter electrocardiography was undertaken for symptomatic events.

### Statistical analysis

The continuous variables were expressed as the mean ± standard deviation or, when indicated, the median and interquartile range. Comparisons between groups were made by a Kruskal-Wallis test for continuous data and a Chi-square test or Fisher's exact test for categorical data. The AT/AF free survival rate of each group was evaluated using the Kaplan-Meier method and compared using the log-rank test. A p value less than 0.05 was considered statistically significant. The analyses were performed using IBM SPSS software version 22.0 (IBM, Armonk, NY, USA).

## CONCLUSION

Creating linear lesions just beneath the neck of the LAA was highly successful under guidance using a circular mapping catheter in the LAA and steerable sheath. An RF application from the CS was needed in less than half of the cases.
